# Long-Term Clinical Outcomes and Patient Satisfaction Following Ultrasound-Guided Hydrodissection for Carpal Tunnel Syndrome: Long-Term Outcomes and Satisfaction after Ultrasound-Guided Hydrodissection for CTS

**DOI:** 10.31661/gmj.v14i.3638

**Published:** 2025-01-20

**Authors:** Ronza R Alsaffar, Zeyad Tareq Kareem, Farah N Abbas

**Affiliations:** ^1^ Physiology Department, Tehran University for Medical Science, Tehran, Iran; ^2^ Alsadiq General Hospital, Babylon Governorate, Iraq; ^3^ Hilla University, Babylon Governorate, Iraq; ^4^ Physiology Department, Babylon University, College of Medicine, Babylon Governorate, Iraq

**Keywords:** Carpal Tunnel Syndrome, Hydrodissection, Ultrasound-Guided Therapy, Median Nerve, Pain

## Abstract

Carpal Tunnel Syndrome (CTS) is popular conditions in where compression of the median nerve causes symptoms such as pain, numbness, and hand weakness in the hand. Current treatments provide varying degree of symptom relief; however, most are limited by short term response or long recovery. Ultrasound-Guided Hydrodissection (USGH) has emerged as a minimally invasive alternative for treating CTS, allowing precise injection of fluid around the median nerve under real-time ultrasound guidance to enhance nerve mobility. Complications of USGH are rare and mild, including short term pain, swelling, or bruising at the injection site. Because of the precision afforded by ultrasound guidance, serious complications, such as nerve damage or infection, are rare. This review aimed to evaluate the long-term clinical outcomes and patient satisfaction after USGH for CTS.

## Introduction

CTS is a neuropathy affecting millions worldwide through the attribution of compression of the median nerve as it passes through the wrist [1][2]. CTS consequently affects hand function and hence one's quality of life, attracting interest in new and long-lasting interventions. The prevalence of CTS and the increasing demand for effective long-term treatments make the search for innovative treatments an important clinical task[3][4]

Current treatments include splinting, corticosteroid injections, and decompressive surgery, and all are limited in their efficacy; furthermore, many offer potential complications [5][6]. Thus, conservative and even surgical treatment often provides short-term alleviation of symptoms [7][8]. This aspect has recently stimulated interest in the employment of relatively harm-free alternatives, such as ultrasound-guided hydrodissection, which have aims at reducing recovery times and eliminating risks related to surgery [8].

Recently, USGH has emerged as a new therapeutic option for CTS. In this technique, fluid-usually saline-is injected into the area of the compressed median nerve under ultrasound guidance as a means of achieving mechanical separation of the nerve from the surrounding tissues and thereby decompression of the nerve [9][10]. This is an attractive option precisely because it is minimally invasive, with presumably quicker recovery than surgical options. Although early studies have reported promising short-term results following USGH, significant long-term efficacy and patient satisfaction remain to be reported and studied [6][11][12].

The objective of this review is to comprehensively consider long-term clinical outcomes regarding USGH for CTS treatment and patient satisfaction, in a presentation of its potential for minimally invasive alternative surgery.

## Pathophysiology of Carpal Tunnel Syndrome

CTS results from compression of the median nerve as it passes through the carpal tunnel, a rigid structure in the wrist formed by the carpal bones and the transverse carpal ligament [13]. The carpal tunnel is bounded by the carpal bones on its floor and sides, and by the transverse carpal ligament on its roof [13][14]. This structure houses the median nerve along with the tendons responsible for finger flexion [13]. Figure 1 illustrates the anatomical structure of the median nerve and the carpal tunnel components.

The median nerve is responsible for sensory and motor functions in the thumb, index, and middle fingers, and when compressed, leads to the classic symptoms of pain, numbness, and tingling. When the median nerve becomes compressed within the confined space of the carpal tunnel, the resulting pressure impairs its function, leading to the hallmark symptoms of CTS [15].

The etiology of CTS is multifactorial. The primary cause is an increase in pressure within the carpal tunnel, which can be triggered by various factors [15][16]. Table-1 presents an overview of the common causes and symptoms of CTS, categorized by occupational, medical, and anatomical factors.

Several factors can increase pressure within the carpal tunnel, including repetitive wrist movements, wrist injuries, or inflammatory conditions [16]. (table 1) Patients with CTS often experience worsening symptoms at night, with the most affected areas being the thumb and first three fingers, as innervated by the median nerve. In advanced cases, there may be visible atrophy of the thenar muscles and a loss of fine motor function, leading to difficulties in performing tasks that require precise finger movements [14][16] (Figure-1).

## USGH Technique

USGH is a minimally invasive procedure designed to relieve median nerve compression in patients with CTS [11]. This procedure involves the precise injection of fluid, typically saline, around the compressed median nerve under real-time ultrasound guidance, allowing for mechanical separation and pressure relief [10].

USGH offers a minimally invasive alternative to corticosteroid injections, which provide temporary results, and surgery, which involves higher risks and longer recovery times [5][6][17]. With ultrasound guidance, USGH allows for faster recovery, sustained symptom relief, and fewer complications [6][17].

This procedure begins with the application of a local anesthetic to the wrist area. A high-frequency ultrasound probe is used to visualize the anatomical structures within the carpal tunnel in real time [6]. Under ultrasound guidance, a fine needle is inserted into the carpal tunnel, and a fluid solution, typically consisting of saline mixed with an anesthetic (and sometimes corticosteroids), is injected [6][11][18]. Also, Wu, et al. [19] and Chen, et al. [20] demonstrated that adding platelet-rich plasma (PRP) to USGH procedure could improve its efficacy.

The fluid works by creating a separation between the compressed median nerve and the adjacent tendons or tissues, a process called hydrodissection. This mechanical release alleviates nerve entrapment and allows for enhanced nerve gliding, which is essential for reducing symptoms of CTS. The procedure usually takes about 15-30 minutes and is performed in an outpatient setting [12].

USGH offers several advantages over corticosteroid injections and surgery, using ultrasound to precisely target the nerve and surrounding structures [17][21]. This reduces the risk of nerve injury, which can occur with blind injections or surgical procedures [5][12]. Additionally, USGH’s minimally invasive nature results in fewer complications, reduced post-procedural pain, lower costs, and faster recovery compared to surgical decompression [5]. This procedure is particularly indicated for patients with mild to moderate CTS who are seeking a less invasive alternative to surgery or for those who have not responded adequately to conservative treatments such as splinting and physical therapy [7][22]. Patients who are unable to undergo surgery due to medical comorbidities, or those who prefer to avoid the risks and extended recovery associated with surgical interventions, may also benefit from USGH [5].

However, certain contraindications should be considered. USGH may not be suitable for patients with severe CTS, where significant nerve damage has already occurred, as these cases often require surgical intervention [12][23]. Morover, patients with local infections at the injection site, allergies to the injectate components (e.g., anesthetics or steroids), or systemic conditions that increase the risk of infection or bleeding may not be ideal candidates for the procedure [6][22].

## Clinical Outcomes of USGH

USGH has gained attention as a minimally invasive treatment for CTS, with both short-term and long-term outcomes being investigated to determine its effectiveness [12]. In the short term, patients frequently notice immediate alleviation of symptoms following the procedure [6]. This is primarily due to the mechanical separation of the median nerve from the surrounding structures, reducing compression and allowing the nerve to glide more freely. Patients typically report reductions in pain, numbness, and tingling, with improved hand function observed within days to weeks after the procedure [7]. The use of anesthetics and, in some cases, corticosteroids during the injection can also contribute to immediate relief by reducing local inflammation [18].

Long-term outcomes, observed after six months or more, are crucial for assessing the sustainability of treatment [24][25]. Extended follow-up studies reveal that most patients maintain symptom improvement, with notable improvements in hand strength, sensory function, and overall QoL [11][22]. However, long-term changes in symptoms may vary depending on the severity of CTS at the time of treatment, along with factors such as age, job demands, and the patient's commitment to post-procedural care recommendations. . In some cases, mild to moderate symptoms can return after a year or more, though re-treatment with USGH may still be possible [22][26]. Table 2 summarizes the clinical studies evaluating the long-term outcomes of USGH in treating CTS.

### Long-Term Clinical Outcomes

Long-term studies confirm that USGH improves pain, functionality, and nerve conduction in CTS patients [22]. For instance, Elawamy et al. [27], eported significant pain reduction, as measured by the Visual Analog Scale (VAS), with the improvement sustained for six months. Similarly, Li et al. [25] found that many patients experienced effective pain relief for over a year. These findings underscore UGHD's ability to provide long-term pain relief, surpassing the short-term benefits of corticosteroid injections.

In addition to pain reduction, improvements in functional disability scores have been consistently reported. Elawamy et al. [27] observed substantial improvements in hand function following USGH, with gains sustained for six months post-procedure. This is supported by Wu et al. [19], who found that patients treated with dextrose hydrodissection showed better functional long-term outcomes.

Also, Long-term follow-up data suggest that UGHD promotes substantial improvements in nerve conduction parameters. Elawamy et al. [27] observed significantly increased sensory nerve conduction velocities and reduced motor latencies at three and six months post-treatment. Similar findings were observed by Li et al., [25] where UGHD treatment led to significant improvements in nerve conduction for CTS patients, with many avoiding surgery even after one to three years of follow-up.

These enhancements in nerve function are critical for restoring sensation and motor control, making UGHD an effective long-term intervention for nerve entrapment conditions.

## Patient Satisfaction and QoL

Patient satisfaction is a key factor in evaluating the efficacy of UGHD [28]. Studies show that UGHD provides significant benefits in terms of patient satisfaction and QoL, with outcomes comparable to more invasive interventions like open surgery [5].

### Patient Satisfaction

Patient satisfaction after UGHD is reported to be notably high, particularly due to its less invasive nature and faster recovery times compared to traditional surgical methods [25]. A comparative study between open surgery and UGHD for treating CTS indicated that both groups of patients experienced significant improvements in symptom severity and functionality, leading to high satisfaction rates in both groups [5]. However, the minimally invasive nature of UGHD tends to reduce postoperative discomfort and rehabilitation time, contributing to higher satisfaction for patients seeking quicker recovery options [27].

Additionally, visual feedback during the procedure enhances patient engagement, further boosting satisfaction. When patients are involved in the process, such as by observing their own ultrasound images during the hydrodissection, their expectations for success and satisfaction with the treatment improve significantly [5].

### QoL Improvements

Studies consistently report improvements in QoL following UGHD, particularly in terms of pain reduction and functional recovery. However, there remains a notable lack of studies that directly measure pre- and post-treatment QoL through commonly used tools such as the 36-Item Short Form Health Survey (SF-36) [29].

In a randomized clinical trial (RCT) assessing the effectiveness of Hyalase hydrodissection, patients treated with UGHD showed significant pain improvement within the first week, with the benefits lasting up to six months. Moreover, their functional disability scores showed marked improvement, reflecting better overall QoL [27].

Another study highlighted that UGHD led to substantial long-term improvements in both nerve function and patient QoL, with over 80% of patients reporting positive outcomes [25]. Studies have also shown that UGHD improves nerve conduction velocity, a key marker of functional improvement in patients with neuropathies [25]. This improvement in nerve function directly correlates with enhanced QoL and functional abilities, allowing patients to return to daily activities more comfortably [27]. Long-term follow-up of patients who underwent UGHD revealed sustained functional improvements and reductions in symptom severity, further emphasizing its efficacy as a treatment option for improving QoL [5].

## Complications and Risks

USGH complications are rare and minor, typically including localized pain, swelling, or bruising that resolve within days [12][23].

Additionally, some patients may experience temporary numbness or tingling, likely caused by transient irritation of the median nerve during fluid injection. These symptoms usually subside as the nerve adapts to the reduced compression [10][12][23].

More serious complications, while rare, can include infection or nerve damage, particularly if proper aseptic techniques are not strictly followed [27].

Though uncommon, inadvertent trauma to the median nerve during needle insertion could theoretically occur, leading to prolonged nerve pain or weakness. However, the real-time visualization provided by ultrasound significantly reduces these risks when compared to blind injection methods [12][22]. Long-term risks are less well-documented. One potential issue is the recurrence of symptoms months or years after the procedure, especially in patients with more severe or chronic CTS [11]. In such cases, repeat hydrodissection or alternative treatments may be required. Although recurrence is not a direct complication of USGH, it underscores the need for further research into the long-term durability of symptom relief [22][25].

Another theoretical long-term risk involves the possible formation of scar tissue at the injection site. Repeated injections into the carpal tunnel could stimulate fibrotic changes, potentially leading to secondary nerve entrapment [25][27]. However, this risk has not been commonly reported in the literature, and further long-term studies are necessary to fully assess this possibility.

## Knowledge Gaps and Future Directions

### Areas for Further Research

While USGH shows significant promise as a minimally invasive alternative for treating CTS, several important knowledge gaps remain. One of the most pressing gaps is the lack of RCTs that directly compare USGH to other established treatment modalities, such as corticosteroid injections and carpal tunnel release surgery [11]. Most current studies are observational, retrospective, or limited in size, which hampers the ability to draw definitive conclusions about USGH’s long-term efficacy and safety. High-quality RCTs are essential to establish stronger, more reliable evidence of its clinical utility [8]. Also, there is a need to investigate the optimal injectate composition and volume for hydrodissection [19][30].

The use of different solutions, such as saline, PRP, or corticosteroids, may lead to variations in clinical outcomes

### Emerging Techniques and Technologies

Looking ahead, advancements in both non-surgical interventions and ultrasound technology hold great potential for improving the treatment of CTS. One key area of development is the refinement of ultrasound guidance techniques [21]. As ultrasound technology continues to improve, with higher resolution and more advanced imaging capabilities, clinicians will be able to perform hydrodissection with greater precision, minimizing risks and improving outcomes [31]. These advances could also enable the treatment of more complex cases, where nerve compression is difficult to visualize or reach using current techniques.

Another promising avenue is the integration of regenerative medicine in USGH procedures. Techniques such as PRP or stem cell injections, in combination with hydrodissection, have the potential to not only relieve symptoms but also promote tissue healing and regeneration [20][32]. By addressing the underlying cause of nerve compression, regenerative therapies may extend the duration of symptom relief and potentially reduce the need for repeat treatments. Preliminary studies suggest that combining regenerative medicine with USGH fosters nerve repair and reduces inflammation, which could lead to better long-term outcomes [19][20].

Moreover, the integration of robotics and artificial intelligence (AI) in chronic pain treatment, including CTS, is another exciting area of research [33][34]. Robotic-assisted ultrasound systems have the potential to improve precision in needle placement and fluid delivery, minimizing variability associated with practitioner skill levels [35]. AI-driven systems could provide real-time feedback, optimizing the hydrodissection procedure and minimizing risks [33].

Non-invasive technologies are also being explored as alternative treatment methods for CTS. Techniques such as focused ultrasound and extracorporeal shockwave therapy (ESWT) aim to relieve nerve compression and reduce inflammation without the need for injections or surgery [36][37]. Although still in the experimental phase, these methods could potentially complement or even replace USGH in select cases, offering non-invasive options for patients who wish to avoid needle-based procedures.

## Conclusion

USGH represents a promising minimally invasive alternative for CTS treatment, which maintains efficacy in the short and long term regarding pain relief, improvement of hand function, and enhancement of nerve conduction, with shorter recovery times. Compared with corticosteroids injections and surgical decompression, USGH has fewer complications and improved recovery time with high rates of patient satisfaction. Also, Real-time visualization during USGH allows for precise targeting and minimizes risks compared to blind injections or surgical approaches.

While USGH has proved especially effective in patients with mild to moderate CTS, further studies are required concerning the long-term effectiveness of this procedure in patients suffering from severe CTS and repeated treatments. Symptom recurrence is another point of interest in ongoing research on the durability of USGH outcomes. Future studies should focus on rigorous RCT execution, establishing superior injectate formulations, and integration of newer developments related to regenerative medicine and robotic-assisted ultrasound for value additions to procedural outcomes. With the advances that continue to envelop ultrasound technology, USGH may eventually assume a typical role in CTS treatment and turn out to be a minimally invasive but fully effective alternative to surgery.

## Conflict of Interest

None declared.
